# Editorial

**Published:** 1902-07-15

**Authors:** 


### EDITORIAL.

WE print in this issue the new law under which Ohio dentists are to practice hereafter. It is in the main a satisfactory law. It seemed necessary to get it enacted, however, to introduce some features which may strike the unsophisticated as peculiar; but fortunately time will eliminate them and the law will apply equally to all. Theoretically such laws are made to protect the innocent public, regardless of individual or even professional interests. In this case it was imperative that the six hundred students enrolled in Ohio dental colleges, as well as the proprietors of illegal and unprofessional dental offices had to be considered, because they had friends in the legislature to look after their interests. They had to be taken care of even to the extent of jeopardizing the constitutionality of the law. The new feature providing for exchange of diplomas with other states of equal standard, will require wise administration to prevent controversies and possible legal embarrassments. The new board just appointed by the Governor is a good one, and we trust the profession will do all that is possible to assist it in putting the new law into effective operation.

OUR two thousand students have graduated from our dental colleges this year. The first question which occurs to us, is, do we need these men to replace those who, from all causes, have retired from practice? The probabilities are that the dental profession does not see any great necessity for such an increase as it means possible interference with established business methods. The people however may be glad that each year brings not only more dentists to look after their teeth, but, that with improved methods and facilities better prepared graduates than ever before are to have charge of their teeth, and as the business competition increases, better service will necessarily come, perhaps not to all; but undoubtedly there will be a larger number of highly qualified practitioners than ever before, and a correspondingly larger number of people will have access to this better service.

The following is a numerical list of the graduates from the several dental colleges for the year:

Atlanta Dental College, 45; Baltimore Dental College, 63; Baltimore Medical College, 26; Birmingham Dental College, 11; Buffalo University, 67; California University, 45; Chicago Dental College, 208; Columbian Dental College, 14; Cincinnati Dental College, 10; Central Indiana Dental College, 16; Colorado Dental College, 24; Des Moines Dental College, 11; Detroit Dental College, 40; Georgetown University, 9; Harvard University, 32; Howard University, 7; Indiana Dental College, 65; Illinois University, 40; Iowa University, 32; Kansas City Dental College, 29; Keokuk Dental College, 13; Lincoln Dental College, 7; Louisville Dental College, 70; Maryland University, 58; Marion Sims Dental College, 36; Maharry Dental College 3; Medico-Chirurgical College, 32; Michigan University, 69; Milwaukee Medical College, 48; Minnesota University, 30; New Orleans Dental College, 17; New York Dental College, 48; New York Dental School, 12; Northwestern University, 163; North Pacific Dental College, 40; Ohio Dental College, 90; Ohio Medical University, 49; Omaha University, 16; Pennsylvania Dental College, 90; Pennsylvania University, 124; Physician and Surgeon of San Francisco, 46; Pittsburg Dental College, 52; Philadelphia Dental College, 114; Richmond University, 12; Royal Dental College, 40; Southern Dental College, 18; Southern California University, 14; Tennessee University, 27; Tuft's College, 32; Vanderbilt University, 18; Western Reserve University, 31; Washington University, 29; Western Dental College, 66. Total, 2,308.

